# Screening the Combination of Gemcitabine, Clomipramine, and Resveratrol in HL-60 Leukemia Cells

**DOI:** 10.3390/cimb48050531

**Published:** 2026-05-19

**Authors:** Burcu Biltekin, Yusuf Elgormus, Ayhan Bilir

**Affiliations:** 1 Department of Histology-Embryology, Istanbul Faculty of Medicine, Istanbul University, 34093 Istanbul, Turkey; ayhan.bilir@atlas.edu.tr; 2 Department of Histology-Embryology, Faculty of Medicine, Istanbul Atlas University, 34403 Istanbul, Turkey; 3 Department of Pediatrics, Istanbul Atlas University Hospital, Faculty of Medicine, Istanbul Atlas University, 34403 Istanbul, Turkey; yusuf.elgormus@atlas.edu.tr

**Keywords:** gemcitabine, clomipramine, resveratrol, HL-60, leukemia, combination therapy, in vitro study

## Abstract

*Background and Objectives*: Potential anti-neoplastic effects of resveratrol, which has antioxidant features combined with clomipramine, which has antineoplastic features, or with gemcitabine, used as a nucleoside analog widely used in chemotherapy, were evaluated together and individually on the HL-60 leukemia cells in this in vitro screening study. *Materials and Methods*: HL-60 cells were treated with gemcitabine, clomipramine, resveratrol, or their combinations at concentrations ranging from 1 to 200 µM. Cell viability was assessed at 24, 48, and 72 h using the trypan blue exclusion method, and results are expressed as a percentage of time-matched untreated controls. Cell proliferation was further evaluated by bromodeoxyuridine (BrdU) immunohistochemical labeling. All experiments were performed in triplicate, and statistical analyses were conducted using one-way analysis of variance (ANOVA) with post hoc comparisons. *Results*: Gemcitabine markedly reduced HL-60 cell viability at all concentrations and time points (*p* < 0.001), indicating strong time-dependent cytotoxicity, with a significant drop in BrdU proliferation index at 48 h (*p* < 0.001). Clomipramine exhibited a biphasic response: high concentrations decreased viability (*p* < 0.05), while low concentrations allowed partial recovery by 72 h. Resveratrol showed concentration-dependent cytotoxicity, with reduced viability at high concentration and near-control levels at low concentration by 72 h; BrdU indices remained significantly lower than control (*p* < 0.001). Combination treatments with gemcitabine showed no additive cytotoxic or antiproliferative effects (*p* > 0.05). A transient enhanced effect was observed in the clomipramine + resveratrol group at 24 h (*p* < 0.01 vs. clomipramine; *p* < 0.05 vs. gemcitabine). *Conclusions*: Gemcitabine, clomipramine, and resveratrol all exhibited inhibitory effects on cell proliferation in HL-60 cell cultures. However, the combination treatments did not show additional cytotoxicity or additive effects. These findings suggest that while each of these compounds individually has the potential to inhibit cell growth, their combined application does not enhance the cytotoxic effects beyond those observed with single treatments. These findings highlight the necessity of a rational approach when considering novel drug combinations.

## 1. Introduction

Malignant diseases attributed to cancer are classically known as diseases with uncontrolled excessive cell proliferation. However, in addition to excessive proliferation, it has been observed that decreased apoptotic cell death rates also contribute to the development of malignancy [1]. Leukemia, one of the most important examples of this, is a malignant formation of hematopoietic stem cells characterized by the replacement of normal cells in the bone marrow by neoplastic cells [2,3].

Gemcitabine (2,2-difluorodeoxycytidine, gemzar) is a deoxycytidine analog nucleoside antimetabolite used in the treatment of various solid tumors [4,5,6]. The in vitro effects of gemcitabine are discussed in studies performed with various solid tumor cell lines [7,8,9]. Despite the known effects of gemcitabine, it is not known exactly how it interacts with tricyclic antidepressant drugs with antineoplastic activities and how it interacts in vitro with resveratrol [10,11,12], a natural polyphenol found in grapes and in significant concentrations in red wine, recognized for its anticancer properties, but its cellular effects are still controversial.

The effects of drugs used in tumor treatment at the cell level have been investigated using many different methods [13]. Many cell culture studies are usually performed using two-dimensional tumor cell cultures. It is known that the results obtained from two-dimensional cell cultures are often not compatible with in vivo conditions. For this reason, the best in vitro models that are compatible with vivo and provide microenvironmental properties in vitro are being studied. The effect of gemcitabine has been investigated with two-dimensional and a few three-dimensional cell cultures in studies conducted with this method to date [14]. Clomipramine, a tricyclic antidepressant with multifaceted effects on the cell cycle, acts through mitochondrial complexes by blocking the oxygen utilization of the cell while performing apoptosis through caspase-3 activation. Resveratrol, on the other hand, induces apoptosis by decreasing Bcl-2 ratios and has multifaceted effects, such as blocking the S phase or affecting the cell by causing DNA damage [15].

The primary motivation for this study is to investigate the cytotoxic effects of resveratrol combined with gemcitabine or clomipramine on HL-60 leukemia cells, both individually and in combinations. Gemcitabine exerts its antiproliferative activity primarily through inhibition of DNA synthesis via ribonucleotide reductase suppression and DNA chain termination [16,17]. Clomipramine acts through a mechanistically distinct pathway, inducing apoptosis via mitochondrial complex III inhibition and subsequent caspase-3 activation [18]. Resveratrol, in turn, operates through yet another non-overlapping mechanism, promoting apoptosis by reducing Bcl-2 expression, inducing S-phase arrest, and causing DNA damage [15]. Given that these three agents target largely independent molecular pathways, their combined application was hypothesized to produce enhanced co-treatment effects through multi-targeted disruption of cell survival, rather than redundant activity along a shared axis.

Previous studies have reported enhanced cytotoxic responses when combining resveratrol with gemcitabine in pancreatic cancer models, attributed to inhibition of the hepatocyte growth factor receptor (c-Met)/poly(ADP-ribose) polymerase 1 (PARP1) resistance axis [16], and when combining clomipramine with imatinib in glioma and with vinorelbine in neuroblastoma models [19,20,21]. However, whether such combined effects translate to a hematopoietic malignancy context, specifically HL-60 acute promyelocytic leukemia cells, remains unexplored. Accordingly, we conducted an equimolar fixed-ratio screening of gemcitabine, clomipramine, and resveratrol in HL-60 cells to evaluate whether co-treatment produces any enhanced antiproliferative effects relative to monotherapy, and to provide a basis for more refined mechanistic studies.

## 2. Materials and Methods

### 2.1. Cell Culture Model

The study was performed under in vitro conditions in the cell culture laboratory of the Department of Histology and Embryology, I.U. Istanbul Faculty of Medicine. HL-60 (CLL-240) cell line obtained from the American Type Culture Collection (ATCC, Manassas, VA, USA) cell bank was used at low passage numbers (<20 passages). Cultures were routinely monitored for contamination and maintained under standard sterile conditions. The media for HL-60 leukemia cells was RPMI 1640 Medium (Gibco™, Thermo Fisher Scientific, Waltham, MA, USA) containing heat-inactivated 10% fetal calf serum (FCS), 100 IU/mL penicillin (Sigma-Aldrich, St. Louis, MO, USA; Cat. No. P-7794), and 100 µg/mL streptomycin (Sigma-Aldrich, St. Louis, MO, USA; Cat. No. S-9137). Cells were grown in 25 cm^2^ and 75 cm^2^ flasks containing this medium in a 37 °C incubator (MCO-18AIC-UV, Sanyo Electric Co., Ltd., Osaka Japan) with 5% CO_2_, 95% air mixture, and humidity, and were routinely passed 3 times a week. A laminar flow cabinet (Tezsan Takım Tezgahları Ltd. Şti., Kayseri, Turkiye) was used for cell passaging, an inverted microscope (Leitz, Wetzlar, Germany) was used for examination of cell cultures, an autoclave (Victor Recker GmbH, Berlin, Germany) was used for sterilization of glassware, and a deep freezer was used for storage of stocks at −20 °C.

### 2.2. Concentration Determination Assay

At the cell collection stage, cells adhering to the upper medium and flask surface were collected and transferred to 15 mL centrifuge tubes. Cells were centrifuged at 1500 rpm for 5 min and suspended in medium, and counted in a hemacytometer. HL-60 cells were seeded at a density of 200,000 cells per well in 6-well culture plates, each containing 2 mL of complete RPMI-1640 medium. Gemcitabine and clomipramine hydrochloride were dissolved in sterile bidistilled water to prepare fresh stock solutions prior to each experiment. Resveratrol (98% purity) was dissolved in dimethyl sulfoxide (DMSO) to prepare a 1 mM stock solution. To ensure complete solubilization, the mixture was vortexed for 2 min and divided into aliquots stored at −20 °C, protected from light to prevent photochemical degradation. Immediately prior to experimental use, the stock was further diluted in RPMI-1640 medium, ensuring that the final DMSO concentration did not exceed 0.1% (*v*/*v*), in accordance with our previously published protocol [22].

Fresh solutions of gemcitabine, clomipramine, and resveratrol at concentrations of 1, 10, 100, and 200 µM each were added to the cells in equal volumes of 100 µL each. We used equimolar fixed-ratio dosing to enable unbiased comparison across arms and to support enhanced co-treatment effects; single-agent concentrations were chosen within literature-supported active ranges. The concentration range (1–200 µM) was selected to cover a broad pharmacological window commonly used in initial in vitro screening studies. This design aimed to identify general cytotoxic trends before conducting more refined dose–response analyses.

Concentration ranges (1–200 µM) were selected from prior literature to cover a broad pharmacological window commonly used in initial in vitro screening studies demonstrating cytotoxicity for each agent in vitro. This design aimed to identify general cytotoxic trends before conducting more refined dose–response analyses. Gemcitabine produces cytotoxic and cell-cycle effects in systems modeling clinical exposures (~15–50 µM in media) and also at lower nanomolar concentrations in certain cell lines when combined with sensitizers (e.g., 100 nM with resveratrol) [20,21]. Resveratrol suppresses proliferation across 0.5–100 µM with model-dependent half maximal inhibitory concentration (IC_50_) values; reports include single-digit micromolar sensitivity as well as higher IC_50_ in metastatic lines [22,23]. Clomipramine induces apoptosis in HL-60 cells via caspase-3 activation at ~35 µM, consistent with a mitochondrial/reactive oxygen species (ROS) mechanism [18]. To compare combinations in a ratio-controlled manner, we employed equimolar dosing as a standard fixed-ratio design used for combination testing. This approach facilitates formal additivity assessment using established methods such as the Chou-Talalay combination index method, in which the combination index (CI) is derived from the median-effect equation and values of CI < 1, CI = 1, and CI > 1 indicate synergism, additivity, and antagonism, respectively [24]. To compare combinations in a ratio-controlled manner, we employed equimolar dosing as a standard fixed-ratio design used for combination testing.

For each concentration of gemcitabine (Gemzar^®^, Eli Lilly and Company, Indianapolis, IN, USA), clomipramine hydrochloride (Turgut İlaç, Istanbul, Turkey) and resveratrol (Bristol-Myers Squibb, New York, NY, USA), 3 wells were seeded. All groups were incubated for 24, 48, and 72 h at 37 °C in a humidified environment with 5% CO_2_ air mixture. After incubation, the cells were collected separately and centrifuged. After discarding the supernatant, they were suspended with 1 mL of medium and proceeded with the trypan blue exclusion assay to determine the effective concentration of gemcitabine, clomipramine, and resveratrol. Total cell counts were determined by counting with a counting chamber (hemacytometer). Live cell numbers were counted for all concentration- and time-dependent assays.

### 2.3. Cell Viability Assay by Trypan Blue Exclusion Assay

HL-60 cells were seeded at a density of 200,000 cells per well in 6-well culture plates, each containing 2 mL of complete medium. The experimental groups included Control, Gemcitabine, Clomipramine, Resveratrol, Gemcitabine + Clomipramine, Gemcitabine + Resveratrol, and Clomipramine + Resveratrol. Cells were treated with the indicated agents and incubated for 24, 48, or 72 h at 37 °C in a humidified atmosphere containing 5% CO_2_. All experiments were performed in triplicate and repeated in three independent experiments to ensure reproducibility of the results.

At the end of each incubation period, cells were harvested by gentle pipetting and collected by centrifugation at 1500 rpm for 5 min. The supernatant was discarded, and the cell pellet was resuspended in 1 mL of fresh medium. Cell viability was assessed using the trypan blue exclusion assay, in which 10 µL of cell suspension was mixed with 10 µL of 0.4% trypan blue solution and incubated for 3 min at room temperature. Viable (unstained) and non-viable (blue-stained) cells were counted using a hemocytometer under an inverted light microscope. The final cell viability percentage was calculated according to the literature as:

Cell Viability (%) = (Number of viable cells/Total number of cells) × 100. The total number of viable cells per well was recorded for each time point and normalized to the time-matched untreated control to express cell viability as a percentage.

IC_50_ values were calculated based on cell number–derived growth inhibition. For each treatment condition, cell numbers were normalized to the corresponding time-matched untreated control and expressed as percentage viability. Dose–response curves were generated by plotting percentage viability against drug concentration, and the IC_50_ was defined as the concentration at which a 50% reduction in cell number was observed relative to control. All analyses were performed using the mean values obtained from independent experiments.

### 2.4. Cell Proliferation Index by Immunohistochemical Labeling with Bromodeoxyuridine

Cell proliferation was determined by immunohistochemical labeling with BrdU according to our protocol previously published [25]. After treatment with the agents for 24, 48, or 72 h, cells were incubated with BrdU (BrdU; Sigma Chemical Co., St. Louis, MO, USA, Cat. No. B-5002) for 60 min at 37 °C before fixation and immunohistochemical staining. Then the cells were rinsed with phosphate-buffered saline (PBS), fixed with 70% ethanol, rinsed with PBS, and smeared on the slides, which were kept in PBS for 20 min. Endogenous peroxidase activity was quenched using H_2_O_2_ for 30 min in the dark and washed 3 times with PBS. After being placed in distilled water and kept in an oven at 37 °C for 5 min, they were kept in 4N HCl for 30 min in an oven at 37 °C. Rinsed with distilled water and washed 3 times with PBS for 5–20 min. Non-specific block followed by 1 h in primary antibody BrdU Ab-4 (Neomarkers, Fremont, CA, USA; Cat. No. 1848P) at room temperature in a humid environment. After washing with PBS, the cells were incubated in secondary antibody (Biotinylated Goat Anti-Mouse, Zymed Laboratories, San Francisco, CA, USA; Cat. No. 13-9669) for 20 min. and kept in streptavidin peroxidase (Zymed Laboratories, San Francisco, CA, USA; Cat. No. 13-9669) for 20 min. After washing with PBS, they were kept in the dark for 20 min in substrate-chromogen (AEC Substrate System, Zymed Laboratories, San Francisco, CA, USA; Cat. No. 00-2007) and after washing with distilled water, they were ground and stained with Mayer Hematoxylin for 15 min. The slides were placed in pure water and covered with a mounting medium (Ultramount, Zymed Laboratories, San Francisco, CA, USA; Cat. No. 00-8000). BrdU-labeled cells in S phase had red-stained nuclei, and at least 100 cells were counted from three regions in each slide for calculation of the BrdU labeling index according to the literature [26]. For negative controls, adjacent sections were processed, excluding the primary antibody.

### 2.5. Statistical Analysis

All statistical analyses were performed using GraphPad Prism version 8.0.1 (GraphPad Software, Inc., San Diego, CA, USA). To evaluate differences in cell viability and BrdU labeling index among treatment groups, a one-way analysis of variance (ANOVA) was conducted separately at each time point (24, 48, and 72 h), followed by Tukey’s honest significant difference (HSD) post hoc test for pairwise comparisons between groups. Each time point was analyzed independently, as the primary objective was to determine whether treatment groups differed from the untreated control and from each other within the same incubation period, rather than to assess longitudinal changes over time. This approach was selected because the experimental design involved independent cell culture preparations at each time point rather than repeated measurements on the same biological unit, making a repeated-measures or two-way ANOVA design inappropriate. Homogeneity of variances was assessed prior to ANOVA using both the Brown-Forsythe and Bartlett’s tests. As the two tests yielded concordant results confirming homogeneity of variances across all treatment groups at each time point, the standard one-way ANOVA assumption of equal variances was considered satisfied, and results are reported accordingly. Data are presented as mean ± SD, and statistical significance was set at *p* < 0.05.

## 3. Results

**Figure 1:**
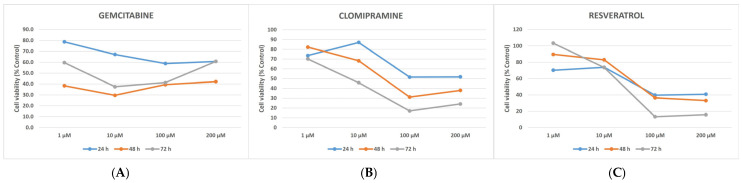
Dose–response effects of gemcitabine (**A**), clomipramine (**B**), and resveratrol (**C**) on HL-60 cell viability at 24, 48, and 72 h. Cell viability is expressed as a percentage of time-matched untreated control values. Concentrations are plotted on a semi-logarithmic scale (1–200 µM). Each data point represents the mean ± SD from three independent experiments performed in triplicate. The dashed horizontal line indicates the 50% viability threshold [22,23].

Figure 1 presents the dose–response curves of gemcitabine, clomipramine, and resveratrol on HL-60 cell viability, expressed as a percentage of time-matched untreated controls, across concentrations of 1–200 µM and three incubation periods (24, 48, and 72 h). Gemcitabine (Panel A) produced a relatively flat dose–response profile, with pronounced cytotoxicity evident even at 1 µM, suggesting a concentration-independent but time-dependent mechanism of action. Clomipramine (Panel B) demonstrated a steep concentration-dependent decline in viability, particularly at 72 h, with minimal cytotoxic effect at 1 µM—a pattern consistent with a threshold-dependent mechanism. Resveratrol (Panel C) exhibited a biphasic dose–response profile: at 1 µM, cell viability at 72 h approached or slightly exceeded control levels, while high concentrations (100–200 µM) caused a marked and sustained reduction in viability across all time points.

IC_50_ values were determined based on cell number–derived growth inhibition, calculated by comparing treated groups with time-matched untreated controls. Across all three agents—gemcitabine, clomipramine, and resveratrol—a dose-dependent reduction in HL-60 cell numbers was observed at 24, 48, and 72 h. As illustrated by the dose–response curves in Figure 1, approximately 50% growth inhibition was observed at 100 µM for all three agents across the evaluated time points, as indicated by the 50% viability reference threshold. However, given the non-sigmoidal and biphasic profiles observed for clomipramine and resveratrol, these values should be interpreted as approximate empirical estimates rather than formally fitted IC_50_ parameters in HL-60 leukemia cells.

**Figure 2:**
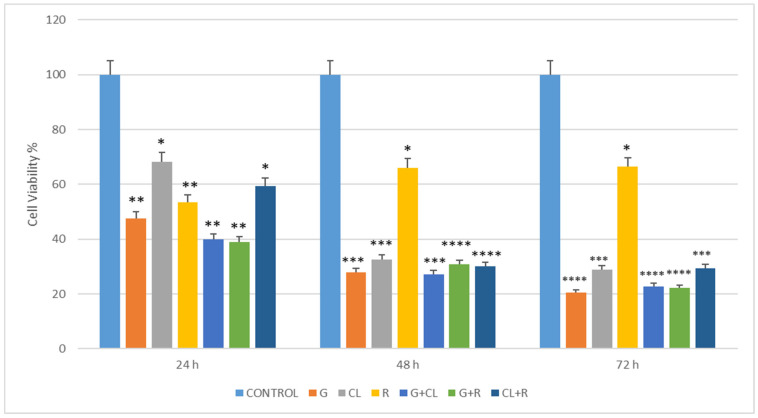
Time-dependent effects of gemcitabine (G), clomipramine (CL), resveratrol (R), and their binary combinations (G + CL, G + R, CL + R) on HL-60 cell viability at 100 µM. Cell viability is expressed as a percentage of the time-matched untreated control group at each time point, calculated as: Cell Viability (%) = (Number of viable cells/Total number of cells) × 100. Cells were incubated for 24, 48, and 72 h under standard culture conditions. Data are presented as mean ± SD from three independent experiments performed in triplicate. Statistical significance was determined by one-way ANOVA followed by post hoc comparisons. Asterisks indicate significance relative to the untreated control group at each time point (* *p* < 0.05, ** *p* < 0.01, *** *p* < 0.001, **** *p* < 0.0001).

Figure 2 presents cell viability as a percentage of the time-matched untreated control for all treatment groups at 100 µM across 24, 48, and 72 h. All treatment groups demonstrated significantly reduced viability compared to the untreated control at each time point. Gemcitabine produced the most sustained reduction, with viability falling to approximately 48%, 28%, and 21% of control at 24, 48, and 72 h, respectively. Clomipramine showed a more moderate reduction at 24 h (approximately 68% of control) with progressive decline at later time points. Resveratrol maintained relatively higher viability at 24 h (approximately 54% of control) compared to gemcitabine and clomipramine, consistent with its biphasic dose–response profile described in Figure 1. None of the combination groups demonstrated viability values lower than those of the most cytotoxically active single agent in each respective pair at any time point, indicating the absence of enhanced combinatory effects under the equimolar conditions tested.

**Figure 3:**
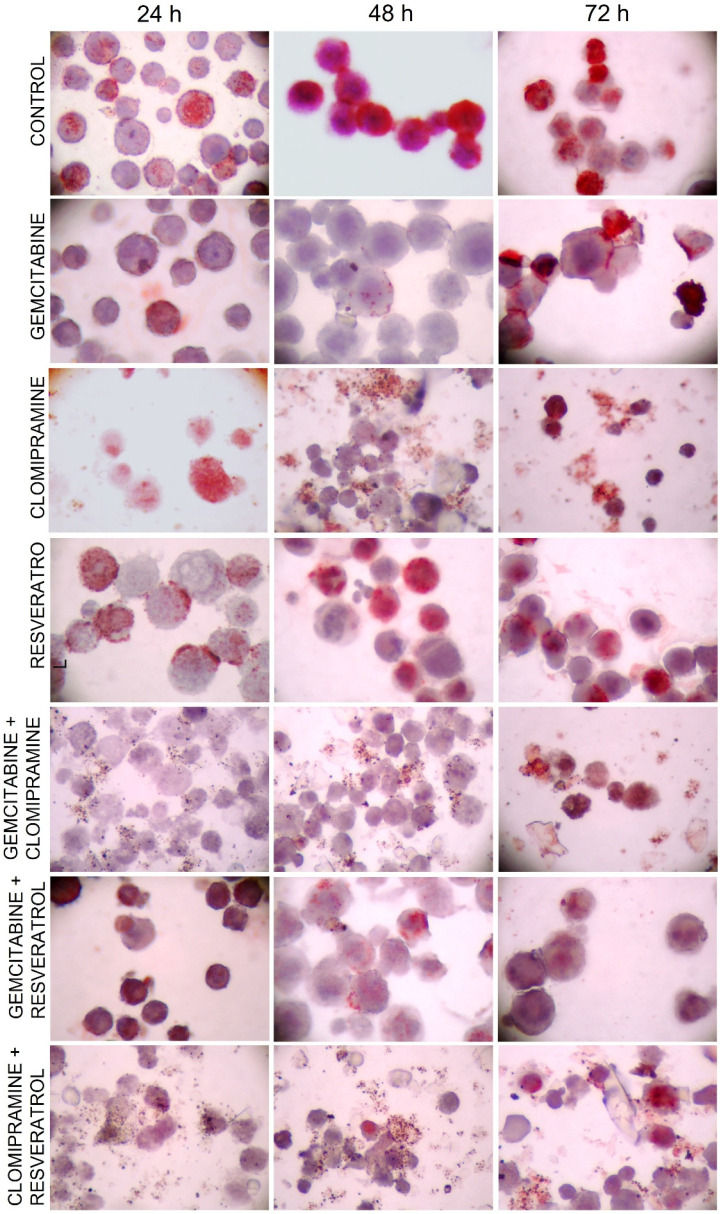
Representative color micrographs showing BrdU immunohistochemical staining in HL-60 cells following treatment with gemcitabine, clomipramine, resveratrol, and their combinations at different time points. BrdU-positive nuclei appear as red staining, indicating cells in the S phase of the cell cycle. Slides were counterstained with hematoxylin. Images were captured using bright-field microscopy at ×400 magnification. Variations in cell size reflect differences in cell cycle stage, treatment-induced cytotoxic effects, and smear preparation characteristics of suspension cells.

Figure 3 presents representative BrdU immunohistochemical staining images at ×400 magnification. In the control group, a high number of BrdU-positive nuclei (red-stained) were evident at all time points, indicating active DNA synthesis and proliferation. Gemcitabine-treated cells showed markedly reduced BrdU staining at 24, 48, and 72 h. In the clomipramine and resveratrol groups, BrdU labeling was also reduced compared to control, but the suppression was less severe than with gemcitabine. Combination groups, particularly those involving gemcitabine, exhibited overall low BrdU labeling, reflecting sustained inhibition of proliferation. The clomipramine + resveratrol group showed a sharp reduction in BrdU positivity at 24 h, but a partial recovery was visible at later time points.

These qualitative findings were quantified in Table 1. The BrdU labeling index was calculated as the percentage of BrdU-positive (red-stained) nuclei among at least 100 cells counted per slide, as described in Methods, and serves as a direct measure of the proportion of cells actively synthesizing DNA at the time of BrdU incorporation. The control group exhibited high proliferation indices at all time points (67.78 ± 1.93 at 24 h, 77.12 ± 1.87 at 48 h, and 74.24 ± 1.31 at 72 h). Gemcitabine treatment caused the most pronounced suppression, particularly at 48 h (6.48 ± 5.78), with statistical significance versus control (*p* < 0.001). Clomipramine and resveratrol both significantly reduced BrdU labeling compared to control (*p* < 0.001), though to a lesser extent than gemcitabine. The clomipramine + resveratrol combination had a significantly lower BrdU index at 24 h (18.89 ± 1.93) than either single agent (*p* < 0.01 vs. clomipramine; *p* < 0.05 vs. gemcitabine), indicating an early enhanced effect that weakened at 48 and 72 h.

Statistical analysis revealed significant differences among all treatment groups at each evaluated time point (*p* < 0.001). Collectively, the viability and proliferation findings indicate that gemcitabine exhibits a pronounced, time-dependent antiproliferative activity; clomipramine and resveratrol demonstrate concentration- and time-dependent effects; and their combined administration leads to an early yet short-lived additive cytotoxic response.

**Figure 4:**
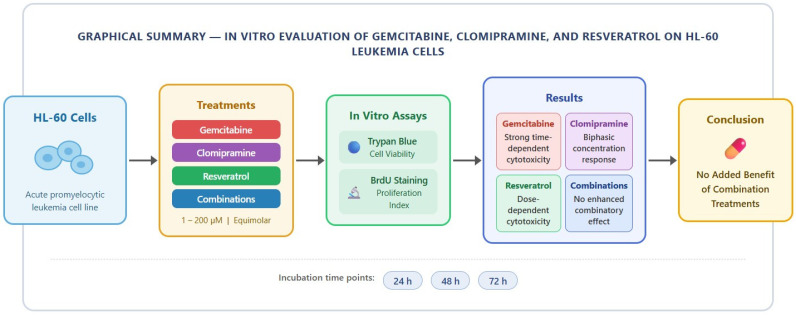
Summary of in vitro evaluation of gemcitabine, clomipramine, and resveratrol, alone and in combination, on HL-60 cell viability and proliferation.

A summary of the experimental design and principal findings is illustrated in the graphical abstract. Gemcitabine showed pronounced time-dependent cytotoxicity, while clomipramine and resveratrol demonstrated concentration-dependent effects. No additive or combinatory effects were observed with combination treatments (Figure 4).

## 4. Discussion

The most important finding of this study is that while gemcitabine demonstrated a strong and sustained antiproliferative effect on HL-60 leukemia cells, the combinations with clomipramine or resveratrol did not consistently enhance this cytotoxicity. Notably, although all three agents individually reduced cell viability and proliferation in a concentration- and time-dependent manner, their combined administration failed to produce a durable combinatory effect. In the clomipramine + resveratrol group, a significant reduction in cell viability and BrdU labeling index was noted at 24 h relative to monotherapy; however, this difference was not observed at 48 or 72 h. Given that this finding was restricted to a single time point and is unsupported by mechanistic data, it should be interpreted cautiously and does not constitute evidence of a sustained or biologically meaningful combinatory interaction. Overall, these screening-level findings confirm the individual antiproliferative activity of each agent without demonstrating durable augmentation beyond monotherapy under the equimolar conditions tested.

The acute promyelocytic cell lineage, associated with acute promyelocytic leukemia, represents one of the most aggressive and lethal blood cancers in humans. The HL-60 cell line, derived via leukapheresis from the peripheral blood of a 36-year-old Caucasian woman with acute promyelocytic leukemia by Collins et al. [14], has become a widely accepted in vitro model for studying this disease. These cells possess the ability to undergo spontaneous differentiation, which can also be induced by agents such as butyrate, hypoxanthine, phorbol myristate acetate, dimethyl sulfoxide (DMSO), actinomycin D, and retinoic acid [15]. The HL-60 is considered a suitable model cell line for studying experimental leukemia types due to its consistent exponential growth, and a relatively short proliferation/differentiation time (PDT) of 36–48 h [12]. For these reasons, HL-60 cells were selected as the experimental model in the present study.

Gemcitabine is a pyrimidine nucleoside analog antimetabolite that shares structural similarities with deoxycytidine and cytarabine [27,28,29]. It acts by interfering with DNA synthesis, primarily through incorporating into the DNA strand, which leads to chain termination and inhibition of further DNA replication. This mechanism makes it effective in treating various cancers, including lung, pancreatic, and bladder carcinoma. Gemcitabine is particularly well-known for its role in combination therapies to improve outcomes in cancer treatments [30]. In the present study, three concentrations of gemcitabine within the 1–200 µM range, as supported by previous literature [27,28,29,30], were administered to HL-60 cells. A significant concentration-dependent reduction in cell proliferation was observed, aligning with earlier findings [27,28,29,30]. The total cell viabilities at 24, 48, and 72 h were markedly lower compared to all corresponding control groups. Gemcitabine’s cytotoxic effect is mediated through intracellular phosphorylation to its active form, which inhibits key enzymes such as ribonucleotide reductase and DNA polymerase, thereby disrupting DNA replication and ultimately halting cell growth [1,29,30]. These results confirm a relatively flat concentration–response profile of gemcitabine on HL-60 leukemia cells in vitro, with sustained cytotoxicity across all concentrations tested, consistent with a time-dependent rather than concentration-dependent mechanism of action.

The increased cytotoxic effect of gemcitabine is believed to be the result of its progressive intracellular accumulation over time [31,32]. In our study, HL-60 cells were exposed to gemcitabine for periods ranging from 24 to 72 h, which may explain the time-dependent increase in cytotoxicity observed. This agent, known for its phase-specific activity during the cell cycle, can persist within cells for prolonged periods, enhancing its therapeutic impact. Such prolonged intracellular retention is especially beneficial in targeting slowly proliferating tumor cells [29]. The longer the exposure, the greater the likelihood that gemcitabine accumulates to cytotoxic levels, thereby inducing substantial DNA damage and inhibiting cell division. This sustained action underlies gemcitabine’s efficacy against a broad spectrum of malignancies, particularly those with low mitotic indices. The 24–72 h treatment window used in our study appears sufficient for the drug to exert its full antiproliferative effect, emphasizing the importance of exposure duration in maximizing gemcitabine’s therapeutic potential.

On the other hand, the concentration-dependent reduction in cell count observed in our study may, in part, be attributed to gemcitabine’s ability to promote cellular differentiation [33]. Additionally, gemcitabine has been shown to increase the activation of topoisomerase I, an enzyme that leads to DNA breakage, further contributing to its cytotoxic effects [34]. These mechanisms offer additional explanations for the antiproliferative activity in our study.

Gemcitabine’s role in inducing cellular differentiation adds a distinct layer to its cytotoxicity. While it is well-known for its inhibitory effects on DNA synthesis and cell proliferation, emerging evidence has highlighted its potential to induce differentiation in certain cancer cell types, including leukemia [35,36]. Differentiation in this context alters the biological behavior of tumor cells, rendering them less proliferative and more susceptible to the cytotoxic effects of chemotherapeutic agents. As differentiated cells often exhibit reduced capacity for division and survival, this process may enhance the overall therapeutic response. In our study, such differentiation-induced changes could be contributing to the observed decrease in HL-60 cell viability following gemcitabine treatment.

Resveratrol, a compound with diverse molecular and biochemical properties, has been shown to inhibit proliferation in various cancers, including leukemia, prostate, and breast cancers [37,38]. Its well-documented antimitotic, antineoplastic, antioxidant, antiproliferative, and anti-inflammatory effects [39] are linked to alterations in cyclin E, cyclin A, cell division cycle 2 kinase (cdc2), and retinoblastoma protein (Rb) expression, leading to cell cycle arrest at the G2-S transition [40,41,42,43]. These mechanisms make resveratrol a promising anticancer agent. In the present study, different concentrations of resveratrol demonstrated a concentration-dependent effect on cell viability and proliferation. The prolonged exposure to resveratrol has likely contributed to the observed cytotoxicity effects, as its impact may accumulate over time and result in more pronounced effects.

Our findings indicate that resveratrol exerts a complex, concentration-dependent effect on HL-60 cell proliferation. While high concentrations significantly reduced cell viability and proliferation index at all time points, lower concentrations demonstrated a diminished cytotoxic effect over time, with cell viabilities approaching control levels by 72 h. This observation suggests that resveratrol’s inhibitory activity may be transient at low concentrations, potentially due to cellular adaptation or activation of compensatory survival mechanisms. The initial antiproliferative response at 24 h could reflect early disruption of cell cycle progression, which becomes less pronounced with prolonged exposure.

Mechanistically, resveratrol is known to interfere with critical cell cycle checkpoints, particularly at the G1/S and G2/M transitions. At high concentrations, it may induce cell cycle arrest by causing accumulation in these phases, contributing to sustained growth inhibition. In contrast, low-concentration exposure has been associated with increased accumulation in the S phase, potentially allowing partial cell cycle progression and explaining the rebound in proliferation observed in our study. Therefore, the reduced cell proliferation seen at early time points and the partial recovery by 72 h at low concentration may reflect a dynamic shift in cell cycle distribution rather than sustained arrest. These findings reinforce the idea that resveratrol’s effects are not only concentration-dependent but also time-dependent, highlighting the need for carefully optimized dosing strategies when considering its use as an adjunct in leukemia therapy.

Clomipramine has key actions in blocking oxygen utilization in cells through the inhibition of mitochondrial complexes, particularly complex III [44,45]. This inhibition disrupts mitochondrial function, which is essential for energy production and cell survival. As a result, clomipramine impairs cellular respiration, leading to reduced energy availability for vital processes, including proliferation. It has demonstrated efficacy in suppressing cell growth in human leukemia cells [46]. Although the molecular mechanisms underlying its effects on the cell cycle are not yet fully elucidated, clomipramine appears to be a promising therapeutic agent, particularly in the context of malignancy, and its influence on cell cycle regulation warrants further investigation [47,48]. In our study, higher concentrations of clomipramine consistently reduced HL-60 cell viability at all time points, indicating a clear cytotoxic effect. Conversely, lower concentrations allowed partial recovery of cell viability over time, particularly at 48 and 72 h. Where prior studies found durable enhanced cytotoxic responses for clomipramine-based pairs (glioma; neuroblastoma) and for resveratrol + gemcitabine (pancreas) [19,21,26], a significant reduction in cell viability was observed in the clomipramine + resveratrol group at 24 h relative to either agent alone; however, this difference was no longer detectable at 48 or 72 h. This isolated observation at a single time point, in the absence of mechanistic data or a sustained dose–response relationship, does not constitute evidence of a biologically meaningful combinatory effect and should not be interpreted as such. It most likely reflects natural variability in the early cytotoxic response under the specific experimental conditions employed, rather than a true pharmacodynamic interaction between the two agents. No mechanistic conclusions can be drawn from this transient finding without supporting data from apoptosis assays, cell cycle analysis, or molecular biomarker studies.

Our rationale for combining these agents stems from evidence that resveratrol enhances gemcitabine efficacy by overcoming chemoresistance mechanisms, while clomipramine triggers apoptosis via mitochondrial and caspase-dependent pathways [16,18,19]. Thus, the triple combination may provide a broader and more potent anticancer effect by simultaneously targeting DNA replication, apoptosis, and resistance mechanisms. Several studies have specifically reported enhanced cytotoxic effects when combining resveratrol with gemcitabine in solid tumor models, representing the most direct comparisons to the negative combination findings of the present study. Yang et al. demonstrated that resveratrol potentiates the antitumor effect of gemcitabine in pancreatic cancer via downregulation of vascular endothelial growth factor-B (VEGF-B), employing non-equimolar dosing optimized for the pancreatic tumor microenvironment [49]. Similarly, Zhou et al. reported that resveratrol reverses gemcitabine-induced stemness in pancreatic cancer cells through sterol regulatory element-binding protein 1 (SREBP1) inhibition, using concentrations and ratios specifically calibrated to exploit resistance mechanisms prevalent in that model [50]. In lung cancer, Qin et al. observed that resveratrol enhances the anticancer efficacy of gemcitabine via endoglin and extracellular signal-regulated kinase (ERK), signaling pathways, again in a solid tumor context with non-equimolar drug ratios [51]. Critically, all three of these studies were conducted in adherent solid tumor cell lines harboring distinct resistance mechanisms including VEGF-B overexpression, cancer stem cell plasticity, and ERK-driven survival signaling that are not characteristic of the HL-60 promyelocytic leukemia model employed here. Furthermore, the concentration ratios used in those studies were not equimolar; rather, they were optimized empirically for each specific tumor context, which is known to substantially influence combination outcomes. In contrast, our study employed a fixed equimolar ratio across a broad concentration range (1–200 µM) as an unbiased initial screen—a design that, while methodologically rigorous for hypothesis generation, may not capture ratio-dependent effects that only emerge under non-equimolar conditions. Taken together, the discrepancy between our findings and those of the aforementioned studies most likely reflects fundamental differences in tumor lineage, molecular resistance background, and combination ratio design, rather than an inherent incompatibility between these agents.

Based on the concentration-response curves, gemcitabine, clomipramine, and resveratrol were each used at 100 µM for combination experiments, corresponding to empirically observed inhibition levels within the range of 50–90% across the evaluated time points. Our concentrations align with ranges where each agent is active in vitro. Gemcitabine exhibits robust effects under exposure profiles equivalent to ~15–50 µM media concentrations and at 100 nM when combined with resveratrol in sensitive lines [20,21]. Resveratrol inhibits proliferation at 0.5–100 µM across models, with reported IC_50_ values spanning low micromolar to higher micromolar in metastatic cells [22,23]. Clomipramine triggers caspase-3–dependent apoptosis in HL-60 at ~35 µM [18,24]. We acknowledge that clomipramine and resveratrol exhibited non-sigmoidal, biphasic concentration–response profiles, particularly at later time points. In such cases, a conventional IC_50_ derived from sigmoidal curve-fitting may not be mathematically valid. Therefore, the reported IC_50_ values for these agents should be interpreted as approximate effective concentrations at which approximately 50% growth inhibition was observed empirically, rather than as formally fitted parameters. Formal dose–response modeling with denser concentration series will be required in future studies.

Combination effects are frequently ratio-dependent; equimolar dosing is a neutral, reproducible starting point that supports fixed-ratio combination-index analyses and response-surface mapping [52]. We therefore used an equimolar design for the initial screen and plan to evaluate non-equimolar ratios (and formal CI/isobologram analyses) in follow-up mechanistic studies as resources permit. When these agents were applied in combination, no additional cytotoxicity was observed in any of the combination groups, and the obtained cell viability values were comparable across these groups. However, the reduction in cell viabilities at all times remained consistent with those observed in the respective monotherapy groups. In each combination group, cell viability was still significantly lower than that of the control groups, indicating that while the individual drugs retained their cytotoxic effects. These results suggest that while combination treatments did not produce enhanced cytotoxicity, they also did not interfere with the antiproliferative activity of the individual compounds. The absence of an enhanced combined effect in our study warrants careful mechanistic interpretation. Gemcitabine exerts its cytotoxicity primarily through S-phase-specific inhibition of DNA synthesis via ribonucleotide reductase suppression and DNA chain termination. Clomipramine acts through inhibition of mitochondrial complex III, impairing cellular energy metabolism and inducing ROS-mediated apoptosis. Resveratrol operates through multiple partially overlapping pathways, including G1/S and G2/M cell cycle arrest, *Bcl-2* downregulation, and DNA damage induction. Importantly, these mechanisms are not mutually exclusive: mitochondrial dysfunction induced by clomipramine could, in principle, amplify DNA damage-triggered apoptotic signaling initiated by gemcitabine or resveratrol. The fact that no enhanced combined effect was observed in the present study therefore does not necessarily imply fundamental mechanistic incompatibility between these agents. Rather, the negative findings may reflect saturation of cytotoxic pathways at the concentrations employed whereby each agent independently drives cell death to a ceiling beyond which additional combinatory input yields no measurable effect. Alternatively, the negative findings may be specific to the HL-60 cell line context, which differs substantially from the solid tumor models in which combination benefits have been reported. Additionally, it should be acknowledged that our equimolar fixed-ratio design, while appropriate as an initial screen, evaluates only a single combination ratio. Combination effects are frequently ratio-dependent, and a more comprehensive assessment, such as a Latin square design covering a matrix of non-equimolar concentration pairs would be required to formally evaluate the combinatory potential of these agents across a pharmacologically relevant dose space [53]. We therefore temper the conclusion that these agents are mechanistically incompatible, and instead propose that the equimolar co-treatment at the tested concentrations failed to produce enhanced antiproliferative effects under the specific experimental conditions employed, leaving open the possibility that optimized non-equimolar ratios or sequential scheduling may yield different outcomes in future studies.

The present study has several limitations that will be addressed in future work. Cell viability was assessed exclusively by trypan blue exclusion, a method that detects only membrane-compromised cells and cannot distinguish early apoptosis from necrotic cell death or cytostatic effects; a dedicated vehicle control for DMSO was also not included. No molecular mechanisms were investigated in the current screening-level design; future studies will incorporate apoptosis markers such as Annexin V/PI staining, caspase activity assays, and flow cytometric cell cycle analysis, alongside a non-cancerous control cell line to enable selectivity index calculations.

Regarding combination design, the equimolar fixed-ratio approach tests only a single concentration ratio per drug pair, and combinatory activity is well-established to be highly ratio-dependent. While this design is compatible in principle with formal additivity assessment frameworks such as the Chou-Talalay combination index method, in which values of CI < 1, CI = 1, and CI > 1 indicate synergism, additivity, and antagonism, respectively, the four-point concentration range and single equimolar ratio employed here do not fulfill the data density requirements for a statistically valid CI calculation. Future studies will therefore employ a Latin square design covering a matrix of non-equimolar concentration pairs, alongside formal combination index and isobologram analyses, to comprehensively evaluate potential enhanced combinatory effects. Sequential dosing schedules, in which pre-treatment with one agent may sensitize cells prior to the addition of a second, will also be tested. The study was conducted in a single HL-60 cell line; future work will include a broader panel of leukemia subtypes and non-cancerous cell lines to assess generalizability and selectivity.

Additionally, the three agents differ substantially in their clinically reported plasma concentrations: gemcitabine achieves approximately 15–50 µM under standard infusion protocols, whereas clomipramine exhibits clinical plasma levels of only 0.3–1 µM and resveratrol achieves nanomolar concentrations due to poor oral bioavailability. The uniform 1–200 µM equimolar range employed here therefore does not reflect pharmacokinetically relevant clinical exposure ratios, and future studies should adopt pharmacokinetically informed dosing strategies. Finally, only conventional two-dimensional culture conditions were used; three-dimensional spheroid or organoid models will be adopted in future work to better recapitulate the tumor microenvironment and improve translational relevance. These improvements will allow a more robust evaluation of combination effects in future studies.

## 5. Conclusions

In conclusion, gemcitabine, clomipramine, and resveratrol each significantly inhibited HL-60 leukemia cell proliferation in a concentration- and time-dependent manner, with gemcitabine showing the most sustained cytotoxicity. While clomipramine and resveratrol were also effective at higher concentrations, their combination with other agents did not enhance cytotoxic effects, indicating a lack of enhanced combined benefit. These results highlight the need for a mechanistic rationale when designing combination therapies, as ineffective combinations may increase toxicity without improving outcomes. Future research should explore optimized dosing, sequential treatments, and molecular-guided strategies to better evaluate the potential for enhanced combinatory effects in leukemia therapy.

## Figures and Tables

**Table 1:** Cell proliferation indexes of BrdU immunohistochemical staining in time-dependent control, gemcitabine, clomipramine, resveratrol, and combination groups.

Groups	24 h	48 h	72 h
Control	67.78 ± 1.93	77.12 ± 1.87	74.24 ± 1.31
Gemcitabine	16.39 ± 3.76 ^a^	6.48 ± 5.78 ^a,c^	26.11 ± 6.74 ^a^
Clomipramine	50.00 ± 16.67 ^b^	24.39 ± 5.93 ^a^	19.05 ± 8.25 ^a^
Resveratrol	27.38 ± 2.06 ^a,c^	27.86 ± 2.57 ^a,b^	33.33 ± 7.22 ^a^
Gemcitabine + Clomipramine	17.72 ± 8.04 ^a,d^	14.39 ± 2.91 ^a^	30.56 ± 4.81 ^a^
Gemcitabine + Resveratrol	28.97 ± 4.18 ^a,c^	16.20 ± 7.65 ^a^	19.44 ± 17.35 ^a^
Clomipramine + Resveratrol	18.89 ± 1.93 ^a,d^	21.43 ± 6.19 ^a,e^	22.17 ± 7.86 ^a^
*p* value	<0.0001	<0.0001	<0.001

BrdU labeling index (%) = (Number of BrdU-positive cells/Total number of cells counted) × 100. A minimum of 100 cells was counted per slide from three independent regions. Values are mean ± SD (*n* = 3) from three independent experiments performed in triplicate. Statistical comparisons were performed using one-way ANOVA followed by Tukey’s post hoc test, applied independently at each time point (24, 48, and 72 h). The rationale for time point-specific analysis is that cell culture preparations were independent at each time point, precluding repeated-measures analysis. The last row presents the overall one-way ANOVA *p*-value for each time point column, reflecting the probability that at least one group mean differs significantly from the others within that time point. Superscript letters denote the following pairwise comparisons: ^a^: *p* < 0.001 vs. the untreated control group; ^b^: *p* < 0.01 vs. the gemcitabine group; ^c^: *p* < 0.05 vs. the gemcitabine group; ^d^: *p* < 0.01 vs. the clomipramine group; ^e^: *p* < 0.05 vs. the clomipramine group. All comparisons are made within the same time point.

## Data Availability

The original contributions presented in this study are included in the article. Further inquiries can be directed to the corresponding author.

## Abbreviations

**Abbreviation**	**Full Term**
HL-60	Human Leukemia-60 (promyelocytic leukemia cell line)
BrdU	Bromodeoxyuridine
RPMI 1640 Medium	Roswell Park Memorial Institute (RPMI) 1640 Medium
FCS	Fetal Calf Serum
IU/mL	International Units per milliliter
PBS	Phosphate-Buffered Saline
AEC	Aminoethyl carbazole (chromogen used in immunohistochemistry)
SD	Standard Deviation
ANOVA	Analysis of Variance
CO_2_	Carbon Dioxide
DMSO	Dimethyl Sulfoxide
